# Characterization of Lignin Extracted from Willow by Deep Eutectic Solvent Treatments

**DOI:** 10.3390/polym10080869

**Published:** 2018-08-05

**Authors:** Gaojin Lyu, Tengfei Li, Xingxiang Ji, Guihua Yang, Yu Liu, Lucian A. Lucia, Jiachuan Chen

**Affiliations:** 1 State Key Laboratory of Biobased Material and Green Papermaking, Qilu University of Technology, Shandong Academy of Sciences, Shandong 250353, China; leetengfly@gmail.com (T.L.); jxx@qlu.edu.cn (X.J.); liuy@qlu.edu.cn (Y.L.); lalucia@ncsu.edu (L.A.L.); chenjc@qlu.edu.cn (J.C.); 2 Department of Forest Biomaterials, North Carolina State University, Box 8005, Raleigh, NC 27695-8005, USA

**Keywords:** deep eutectic solvent (DES), lignin, DES-lignin, molecular weights, lignin nanoparticles, β-aryl ether bonds

## Abstract

Purity, morphology, and structural characterization of synthesized deep eutectic solvent (DES)-lignins (D_6h_, D_9h_, D_12h_, D_18h_, D_24h_) extracted from willow (*Salix matsudana* cv. Zhuliu) after treatment with a 1:10 molar ratio of choline chloride and lactic acid at 120 °C for 6, 9, 12, 18, and 24 h were carried out. The purity of DES-lignin was ~95.4%. The proportion of hydrogen (H) in DES-lignin samples increased from 4.22% to 6.90% with lignin extraction time. The DES-lignin samples had low number/weight average molecular weights (1348.1/1806.7 to 920.2/1042.5 g/mol, from D_6h_ to D_24h_) and low particle sizes (702–400 nm). Atomic force microscopy (AFM) analysis demonstrated that DES-lignin nanoparticles had smooth surfaces and diameters of 200–420 nm. Syringyl (S) units were dominant, and total phenolic hydroxyl content and total hydroxyl content reached their highest values of 2.05 and 3.42 mmol·g^−1^ in D_12h_ and D_6h_, respectively. β-Aryl ether (β-*O*-4) linkages were eliminated during DES treatment.

## 1. Introduction

As a substitute for fossil energy, biomass-based energy has received significant attention over the last decade [1,2]. Willow (*Salix matsudana* cv. Zhuliu), among the various biomass resources of interest, is a new hybrid plant variety (Salicaceae *Salix* spp.) that displays strong resistance and high survival rate. It is widely planted in desert areas for wind prevention and sand fixation. Moreover, particularly in China with scarce timber resources, it has great potential in pulp and papermaking operations, due to its fast-growing nature [3].

Lignin is one of the three components (cellulose, hemicellulose, and lignin) that make up biomass, and the only fully aromatic polymer found in nature. It is a bio-based raw material attracting intense research in conversion and modification, despite its complex structure [4,5,6]. The effective separation of lignin from lignocellulosic biomass, nonetheless, is key to subsequent utilization. A variety of isolation technologies have been developed to effect its isolation such as alkaline, enzymatic, and dilute acid hydrolysis, organosolv, ionic liquids, and deep eutectic solvent extraction [7,8,9,10,11,12,13].

Deep eutectic solvents (DESs) are a fundamentally new paradigm in the chemical sciences. Because of their highly tunable nature and exceptional properties, DESs have become essential tools within the fields of extraction, analytics, electrochemistry, biotechnology, catalysis, and synthesis amongst various disciplines [14,15,16]. Over the last decade, global research efforts have explored the possibilities for using them as environmentally benign alternatives for positioning lignocellulosic biomass and its derivatives as alternative green feedstocks to petroleum. Numerous research efforts have unearthed dissolution behaviors, processes, catalytic conversions, and modifications of carbohydrates using DESs, while the upgrading of lignin in DESs also promises a variety of chemical opportunities [17,18,19,20,21,22]. The use of DESs as alternative media for lignin extraction and isolation, upgrading, modification, and depolymerization have gained recent attention [22,23,24,25]. Previous studies have found that choline chloride can separate compounds, such as phenol, from solution by forming DESs through interactions with phenolic hydroxyl groups [22]. Recently, DES-based isolation of lignin by cleaving ether linkages has already been explored and showed some unique advantages compared to traditional ionic liquid treatment, such as low cost, non-toxicity, environmental friendliness, etc. [25,26]. Additionally, the lignin extracted by DES treatments has been reported to have high yield, high purity, and low molecular weight, which would be of benefit for future valorization [23]. However, the unique structural properties of lignin isolated by DESs were neither thoroughly studied nor discussed.

In the present examination, the purity, morphology, and structural characteristics of DES-lignin samples, i.e., lignin extracted from willow by DES treatment at a 1:10 molar ratio of choline chloride and lactic acid treatment at 120 °C for varied times, were investigated by acid hydrolysis, elemental analysis, GPC, nanoparticle size, AFM, ^31^P NMR, and 2D-HSQC NMR.

## 2. Materials and Methods 

### 2.1. Materials

A five-year-old willow (*Salix matsudana* cv. Zhuliu) was harvested in a forest center in Dingxi, Gansu province, China. The sample of willow trunk (bark and branches removed, a length of 1.0 m, and diameter cross-section of 22 cm, approximately 17 kg) was air-dried for two months in a laboratory. The air-dried willow was cut to 1–1.5 cm in size and ground in a star mill. The fractions obtained by sifting through a 40–80 mesh sieve were used for the subsequent separation of the DES-lignin. The choline chloride and lactic acid used in the synthesis of the deep eutectic solvent were provided by Shanghai Maclean Biochemical Technology Co., Ltd. (Shanghai, China).

### 2.2. Isolation of DES-lignin

A deep eutectic solvent (DES) composed of choline chloride and lactic acid (molar ratio 1:10) was applied for isolating lignin from willow. Raw material was first extracted with organic solvents (*v*/*v*, benzene to 95% ethanol = 2/1) for 8 h to remove extracts. The extracted sample (2.5 g) was added to a round-bottomed flask containing DES (m/m of raw material to DES = 1 to 30). The mixture of willow sample and DES was heated to 120 °C by an oil bath with magnetic stirring, and treatment temperatures (6–24 h) effects were studied.

After treatment, the DES soluble fractions and solid residues were separated by filtration using a G2 glass crucible. The solid residues were thoroughly washed with anhydrous ethanol. The filtrate was combined and concentrated to recover ethanol, and was then added to deionized water (2000 mL) to precipitate lignin. After 24 h, the precipitate was collected after centrifugation, and washed with an ethanol/water mixture (1:9, *v*/*v*). The DES-lignin or the lignin extracted from willow by DES treatment, was obtained by vacuum freeze-drying [25]. The isolated DES-lignin samples were labeled D_6h_, D_9h_, D_12h_, D_18h_, D_24h_ (the DES-lignin extracted from willow by DES treatment for 6, 9, 12, 18, and 24 h).

### 2.3. Willow Enzymatic/Mild Acidolysis Lignin (EMAL) Preparation

The 40 to 60 mesh fractions of willow after acetone extraction for 48 h were used as raw material for the lignin separation. The dried raw material was placed in a roller ball mill (PM200, Retsch, Düsseldorf, Germany) for 72 h at a rotation frequency of 500 rpm. The ball-milled raw material was used for the preparation of EMAL after being subjected to ethanol and benzene extraction for 8 h. The preparation of EMAL was based on past research [3].

### 2.4. Purity Analysis of DES-lignin

The chemical composition of the DES-lignin samples was analyzed by NREL methods [27], and the purity of DES-lignin was calculated from the sum of acid insoluble lignin (%) and acid soluble lignin (%).

### 2.5. Elemental Analysis of DES-lignin

The carbon (C), hydrogen (H), nitrogen (N) contents (wt %) in the DES-lignin samples were analyzed by the elemental analyzer (Vario EL III, Elementar, Frankfurt, Hesse, Germany). The oxygen content (wt %) was determined by difference.

### 2.6. Molecular Weight Distribution Analysis of DES-lignin and EMAL

The molecular weight distribution of the DES-lignin and EMAL samples were identified using liquid gel permeation chromatography (GPC) 1200 (Agilent Technologies Inc., Palo Alto, CA, USA). Ten milligrams of each sample were completely dissolved in 10 mL high-performance liquid chromatography-grade THF, and 20 μL of resulting solutions were injected into the column. The eluent (THF) flow rate was kept as 1.0 mL/min, and the temperature was maintained at 30 °C. A set of polystyrene standards (200 to 30,000 Da, Aldrich, Shanghai, China) were used for calibration.

### 2.7. Particle Size Analysis of DES-lignin

A nanoparticle size analyzer (Zetasizer Nano S90, Malvern Instruments Ltd., London, UK) was used to measure the particle size of DES-lignin samples. Samples (10 mg) were dissolved in 10 mL THF with ultrasound, from which a 1 mL solution was added to a quartz cuvette for analysis. The sample was circulated three times for a total of 10 min to identify the average size for each sample.

### 2.8. Atomic Force Microscope (AFM) Analysis of DES-lignin

The DES-lignin sample was completely dissolved in acetone at a concentration of 0.1 g per liter, and a drop of diluted DES-lignin suspension was deposited onto a clean mica surface and air-dried overnight in ambient environment before examining the DES-lignin morphologies using atomic force microscope (AFM) imaging (AFM Workshop). The morphologies of DES-lignin sample were analyzed using the NanoScope analysis software (Bruker, Karlsruhe, Germany) with the AFM system. The resolution in the X-axis and Y-axis direction is 1 nm, and the resolution in the Z-axis direction is 0.1 nm.

### 2.9. ^31^P NMR Analysis of DES-Lignin

DES-lignin samples (25 mg) were completely dissolved in 400 μL deuterated chloroform and deuterated pyridine (1:1.6, *v*/*v*). Then, cyclohexanol (4 mg·mL^−1^) and chromium(III) acetylacetonate (3.6 mg·mL^−1^) were added into the solution as internal standard and relaxation reagent, respectively. The mixed solution was reacted with 75 μL of phosphating reagent (2-chloro-4,4,5,5-tetramethyl-1,3,2-dioxaphospholane, TMDP) for 10 min, and transferred into a 5 mm NMR tube for ^31^P NMR analysis on a 400 MHz Bruker AVANCE III 400 spectrometer (Bruker, Karlsruhe, Germany).

### 2.10. 2D-HSQC NMR Analysis of DES-Lignin and EMAL

Approximately 80 mg samples and 500 μL of dimethyl sulfoxide (DMSO)-*d*_6_ were stirred at 25 °C for 30 min to obtain a solution. The solution was then analyzed by using an ADVANCE III 600 MHz spectrometer (Bruker, Karlsruhe, Germany) for 16 h. Matrices of 1024 data points for the ^13^C dimension and 2048 data points for the ^1^H dimension were collected from 160 to 0 ppm and 13 to −1 ppm for the ^13^C and ^1^H dimensions, respectively. Relaxation delay was set at 6 s.

## 3. Results and Discussion

### 3.1. Purity Analysis

To investigate the purity of DES-lignin samples (D_6h_, D_9h_, D_12h_, D_18h_, D_24h_), the acid soluble lignin (ASL), acid insoluble lignin (AIL), sugars, and ash content in DES-lignin samples were measured (Table 1). The purity of all lignin samples was >90%, and with extension of treatment time, the purity increased from 90.0% to 95.4%. This showed that 1.12% of glucose, 0.96% of xylose, 0.12% arabinose and 0.05% mannose were present in D_6h_, which may be attributed to the presence of LCCs (lignin–carbohydrate complexes) from the plant cell wall [28,29,30]. It was interesting that only 0.21% of glucose and 0.15% of xylose were present in D_12h_, suggesting that less carbohydrate remained in the DES-lignin at an extraction time of 12 h relative to 6 h, which may be the reason for the increased purity. The monosaccharides were not detected in the acid hydrolysis of D_24h_, indicating that the residual carbohydrates in the separated lignin were negligible when extraction time was >24 h.

### 3.2. Element Analysis

The elemental compositions of the DES-lignin samples (D_6h_, D_9h_, D_12h_, D_18h_, D_24h_) from DES treatments are listed in Table 2. DES-lignin had a high carbon content (58.4–60.0%), and negligible amount of nitrogen (0.39–0.45%). With extraction time, the content of oxygen in the separated DES-lignin samples gradually decreased from 35.87% in D_6h_ to 33.26% in D_24h_. The hydrogen content increased from 4.22% in D_6h_ to 6.90% in D_24_, which may mean that the oxygen-containing functional groups or linkages, such as methoxy group and aryl ether bond in willow lignin, were eliminated during DES treatment. It has been previously proposed that both the cleavage of ether bonds and the solvent properties of DES played an important role on the depolymerization of lignin, which facilitated its separation from wood [23]. DES may provide a mild acid–base catalysis mechanism that would favorable for cleavage of labile ether linkages, and thus lead to generation of a low molecular weight lignin fragment [31]. This chemistry may affect the elemental composition of the isolated lignin.

### 3.3. Molecular Weight Distribution

**Figure 1:**
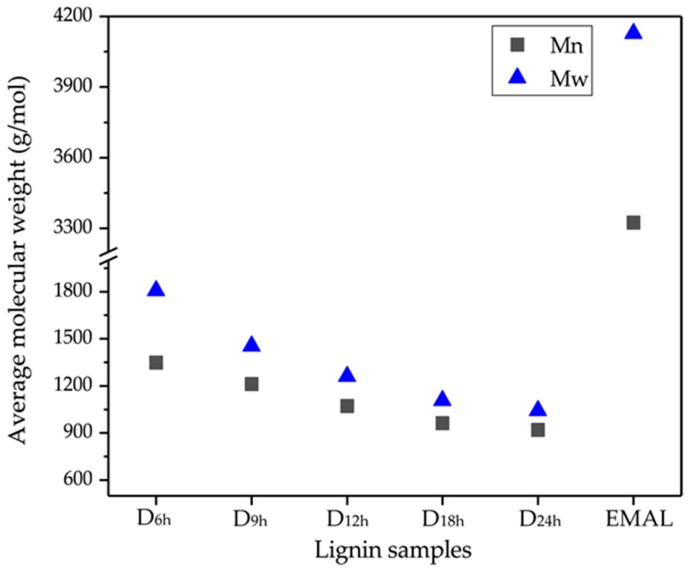
Number average molecular weight ($\bar{Mn})$ and weight average molecular weight $\left(\bar{Mw}\right)$ of DES-lignin samples (D_6h_, D_9h_, D_12h_, D_18h_, D_24h_) from willow by DES treatment at 120 °C for 6, 9, 12, 18, and 24 h. The EMAL was extracted from willow by enzymatic/mild acid hydrolysis treatment.

The change of number average molecular weight ($\bar{Mn})$ and weight average molecular weight $\left(\bar{Mw}\right)$ distribution of DES-lignin samples (D_6h_, D_9h_, D_12h_, D_18h_, D_24h_) are shown in Figure 1. In addition, the values of the $\bar{Mn}$ and $\bar{Mw}$ from the GPC curves and the polydispersity index ($\bar{Mw}$/$\bar{Mn}$) are listed in Table 3. Compared with EMAL and lignin extracted by other methods, the number average and weight average molecular weight of DES-lignin were significantly reduced [3,32,33]. The $\bar{Mw}$ and $\bar{Mn}$ of DES-lignin samples extracted from willow by DES treatment at 120 °C at different times decreased from 1806.7 and 1348.1 g/mol of D_6h_ to 1042.5 and 920.2 g/mol of D_24h_, which indicated that the average molecular weight of DES-lignin gradually decreased with increase of DES treatment time. The above results may be due to elimination of intermolecular linkages of lignin under DES, and with increase of time, the degradation of the lignin macromolecule was more significant, similar to traditional ionic liquids [10,34].

The polydispersity of DES-lignin samples and EMAL was calculated. It can be seen from Table 3 that the polydispersity value of DES-lignin samples decreased from 1.34 of D_6h_ to 1.13 of D_24h_, which indicated that the distribution of DES-lignin gradually concentrated with treatment time. The polydispersity of the lignin extracted from willow by DES treatment was lower than EMAL when the DES treatment time was >9 h.

### 3.4. Particle Size Analysis

The lignin particle sizes by DES treatment are shown in Table 4. The particle size of all the DES-lignin samples were lower than 1 μm and the particle size of DES-lignin samples decreased substantially from 704 to 400 nm with extraction time from 6 to 18 h. However, D_18h_ and D_24h_ had nearly the same particle size (~400 nm), which suggested that it was difficult to further reduce lignin particle size with more treatment time.

### 3.5. AFM Analysis of DES-lignin

**Figure 2:**
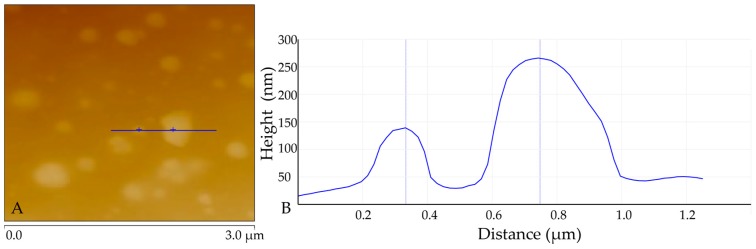
AFM analysis image of DES-lignin sample (D_12h_) extracted from willow by DES treatment at 120 °C for 12 h. (**A**): height mode AFM image; (**B**) AFM-measured topographical height profiles corresponding to the line in (A).

The topic of lignin nanoparticles for green biobased functional materials is becoming more and more appealing [35,36]. Morphological regularity and small particle sizes are the key criteria for the successful preparation of lignin-based functional materials. To investigate the surface morphology of DES-lignin, the DES-lignin sample (D_12h_) was dissolved in acetone at a concentration of 0.1 g per liter, after which a drop of liquid was air-dried and examined on clean mica by atomic force microscopy (AFM). Figure 2 confirmed that nanoscale lignin particles formed by self-assembly of dissolved lignin after precipitation via dilution. Aggregated particle indicated by multiple peaks in profile in Figure 2B, corresponding to the line in Figure 2A with diameters between 200 nm to 420 nm, were observed. There were broad peaks in the smooth curve of Figure 2B, which indicated that the surface of DES-lignin nanoparticles was relatively smooth.

### 3.6. ^31^P NMR Analysis of DES-lignin

**Figure 3:**
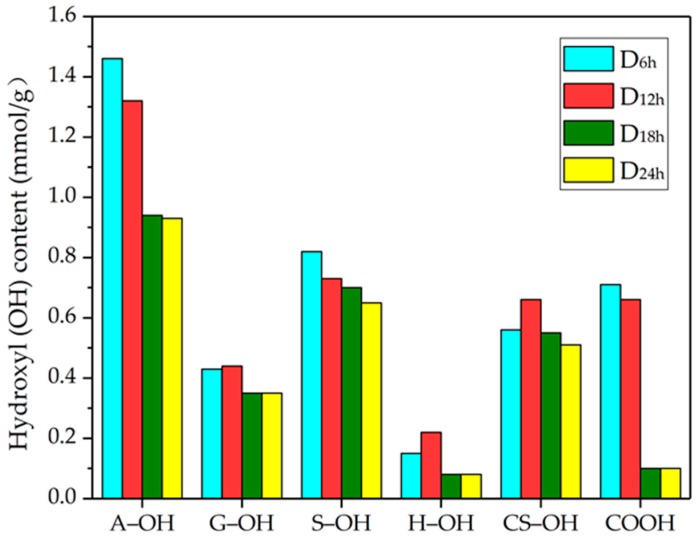
Quantification of different hydroxyl group (A–OH: aliphatic hydroxyl; G–OH: guaiacyl phenol hydroxyl; S–OH: syringyl phenol hydroxyl; H–OH: *p*-phenol hydroxyl; CS–OH: condensation phenol hydroxyl; COOH: carboxyl) contents (mmol/g) of DES-lignin samples (D_6h_, D_12h_, D_18h_, D_24h_) extracted from willow by DES treatment at 120 °C for 6, 12, 18, and 24 h as determined by ^31^P NMR.

^31^P NMR can be used to quantitate hydroxyl groups on lignin [37,38]. In Figure 3, lignin extracted from willow by DES treatment is GSH (containing guaiacyl, syringyl, and *p*-hydroxyphenyl units) type lignin, and the syringyl units were dominant, consistent with the structure of hardwood lignin [39,40]. The content of the guaiacyl phenol hydroxyls (G–OH) and *p*-phenol hydroxyl (H–OH) of D_12h_ was highest compared to D_6h_, D_18h_, and D_24h_. The content of the syringyl phenol hydroxyl (S–OH) decreased from 0.82 mmol·g^−1^ in D_6h_ to 0.65 mmol·g^−1^ in D_24h_, indicating the content of S–OH in DES-lignin extracted by DES gradually decreased with treatment time (see Table 5). Generally, the content of total phenol hydroxyl reached its highest value for D_12h_, and the content of total hydroxy groups in D_6h_ was the highest mainly due to the presence of aliphatic hydroxy (A–OH) in D_6h_ relative to other DES-lignin samples. Interestingly, the content of A–OH in the extracted DES-lignin samples decreased from 1.46 mmol·g^−1^ in D_6h_ to 0.93 mmol·g^−1^ in D_12h_ when the extraction times of DES-lignin increased from 6 to 24 h, which may be due to gradual decrease of the long carbon chains in the separated lignin and the carbohydrates attached to the lignin under the treatment of DES for a longer period of time [41,42]. It can also be seen from Figure 3 that the content of the carboxyl (COOH) in the extracted lignin was significantly reduced when the extraction time was extended to 18 h.

### 3.7. 2D-HSQC NMR Spectra of DES-lignin and EMAL

**Figure 4:**
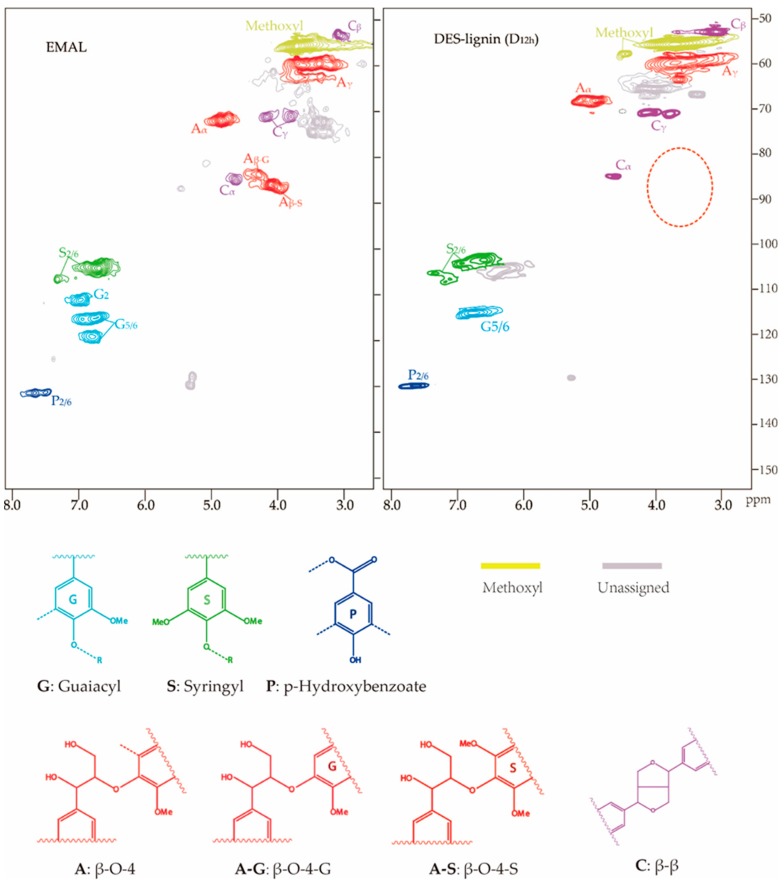
2D-HSQC NMR analysis of DES-lignin sample (D_12h_) extracted from willow by DES treatment at 120 °C for 12 h and the EMAL extracted from willow by enzymatic/mild acid hydrolysis treatment.

To investigate the structural characteristics of DES-lignin, 2D-HSQC NMR for DES-lignin sample (D_12h_) extracted from willow by DES-treatment at 120 °C for 12 h and the EMAL of willow were obtained (Figure 4). Assignment of the peaks in the 2D-HSQC NMR spectra was conducted based on previous reports [32,43,44,45]. The peaks corresponding to methoxyl (3.81/56.7 parts per million (ppm)) and β-aryl ether are shown for A_α_ (4.91/70.3 ppm) and A_γ_ (4.05/87.5 ppm), β–β are shown for C_α_ (4.71/86.1 ppm), C_β_ (3.08/53.9 ppm) and C_γ_ (4.11/70.3 ppm), guaiacyl (G_5/6_, 6.79/115.1 ppm), syringyl (S_2/6_, 6.61/102.9 and 7.19/116.2 ppm), and *p*-hydroxybenzoate (P_2/6_, 7.61/131.2 ppm) appeared in the DES-lignin (D_12h_) NMR spectrum indicating functional groups associated with lignin were present and not severely damaged during DES treatment. Correlations for the syringyl β-aryl ether corresponding to A_β-S_ (4.05/87.5 ppm), and the guaiacyl β-aryl ether corresponding to A_β-G_ (4.23/83.5 ppm) were absent, suggesting that the β-aryl ether linkages in willow lignin were eliminated during DES treatment, similarly to the effect of ionic liquids on lignin [11,12,46]. From Figure 4, the correlation for the guaiacyl corresponding to the G_2_ (6.97/110.7 ppm) and G_5/6_ (6.83/118.9 ppm) disappeared in the DES-lignin spectrum compared to the EMAL spectrum, which may indicate that part of guaiacyl in willow lignin was eliminated during DES treatment.

## 4. Conclusions

High purity (90–95.4%) lignin can be obtained from willow (*Salix matsudana* cv. Zhuliu) after DES (the molar ratio of choline chloride to lactic acid was 1 to 10) treatment. The elemental composition of DES-lignin varies with the extraction time. The DES-lignin had low molecular weights and low particle sizes, and were reduced with extension of the extraction time. Atomic force microscopy (AFM) analysis results demonstrated that the lignin nanoparticles extracted from willow by DES treatment had smooth surfaces and diameters of 200–420 nm. DES-lignin is discernible as GSH lignin, and the syringyl (S) units were dominant while the total phenolic hydroxyl content and total hydroxyl content reached their highest values (2.05 and 3.42 mmol·g^−1^) in D_12h_ and D_6h_, respectively. In addition, the β-aryl ether (β-*O*-4) linkage was eliminated during DES treatment.

## Figures and Tables

**Table 1:** Purity of the DES-lignin samples (D_6h_, D_9h_, D_12h_, D_18h_, D_24h_) extracted from willow by DES treatment at 120 °C for 6, 9, 12, 18, and 24 h.

Component	D_6h_ (%)	D_9h_ (%)	D_12h_ (%)	D_18h_ (%)	D_24h_ (%)
Acid insoluble lignin (AIL)	88.54	90.86	92.57	93.45	93.69
Acid soluble lignin (ASL)	1.48	1.51	1.89	1.63	1.71
Glucose	1.12	0.77	0.21	0.11	-
Xylose	0.96	0.56	0.15	-	-
Arabinose	0.12	-	-	-	-
Galactose	0.05	-	-	-	-
Mannose	-	-	-	-	-
Ash	0.51	0.60	0.51	0.54	0.59
Lignin purity *	90.02	92.37	94.46	95.08	95.40

* Lignin purity is calculated from the sum up of AIL (%) and ASL (%) of willow.

**Table 2:** Elemental analysis of DES-lignin samples (D_6h_, D_9h_, D_12h_, D_18h_, D_24h_).

DES-Lignin Samples	Elemental Analysis (wt %)				Element Molar Ratio	
	C	H	N	O	O/C	H/C
D_6h_	59.01	4.22	0.39	35.87	0.46	0.86
D_9h_	59.42	4.78	0.45	34.75	0.44	0.97
D_12h_	60.08	5.50	0.41	33.50	0.42	1.10
D_18h_	59.59	6.04	0.43	33.40	0.42	1.22
D_24h_	58.84	6.90	0.41	33.26	0.42	1.42

**Table 3:** Average molecular weight and polydispersity of DES-lignin samples (D_6h_, D_9h_, D_12h_, D_18h_, D_24h_) and EMAL. ($\bar{Mn}$): number average molecular weight which can be averaged according to the number of molecules; ($\bar{Mw}$): weight average molecular weight which can be averaged according to the weight of molecules; $\bar{Mw}$/$\bar{Mn}$: polydispersity.

Lignin Samples	$\bar{Mn}\;(g/\mathrm{mol})$	$\bar{Mw}\;(g/\mathrm{mol})$	$\bar{Mw}$/$\bar{Mn}$
D_6h_	1348.1	1806.7	1.34
D_9h_	1212.3	1454.2	1.20
D_12h_	1071.1	1261.1	1.18
D_18h_	962.9	1106.3	1.15
D_24h_	920.2	1042.5	1.13
EMAL	3324.9	4127.0	1.24

**Table 4:** Particle size of DES-lignin samples (D_6h_, D_9h_, D_12h_, D_18h_, D_24h_) extracted from willow by DES treatment at 120 °C for 6, 9, 12, 18, and 24 h.

DES-Lignin Samples	D_6h_	D_9h_	D_12h_	D_18h_	D_24h_
Particle size (nm)	704.7	612.4	494.2	400.1	402.4

**Table 5:** ^31^P NMR analysis of DES-lignin samples (D_6h_, D_12h_, D_18h_, D_24h_) extracted from willow by DES treatment at 120 °C for 6, 12, 18, and 24 h.

Chemical Shift (ppm)	Assignment	Content (mmol·g^−1^)			
		D_6h_	D_12h_	D_18h_	D_24h_
133.7–134.9	Carboxyl (COOH)	0.71	0.66	0.10	0.10
136.9–138.6	*p*-Ohenol hydroxyl (H–OH)	0.15	0.22	0.08	0.08
138.6–140.3	Guaiacyl phenol hydroxyl (G–OH)	0.43	0.44	0.35	0.35
142.3–143.8	Syringyl phenol hydroxyl (S–OH)	0.82	0.73	0.70	0.65
-	G:S:H	2.9:5.5:1	2.0:3.2:1	4.4:8.8:1	4.4:8.1:1
140.3–142.3 143.8–144.5	Condensation phenol hydroxyl (CS–OH)	0.56	0.66	0.55	0.51
145.2–150.2	Aliphatic hydroxyl (A–OH)	1.46	1.32	0.94	0.93
	Total phenol hydroxyl	1.96	2.05	1.68	1.59
	Total hydroxyl	3.42	3.37	2.62	2.52

## References

[1] Junior H.J.E., de Melo R.X., Sartori M.M.P., Guerra S.P.S., Ballarin A.W. (2016). Sustainable use of eucalypt biomass grown on short rotation coppice for bioenergy. Biomass Bioenergy.

[2] Lin X., Wu Z., Zhang C., Liu S., Nie S. (2018). Enzymatic pulping of lignocellulosic biomass. Ind. Crop. Prod..

[3] Guo T., Liu Y., Liu Y., Yang G., Chen J., Lucia L.A. (2016). Chemical elucidation of structurally diverse willow lignins. BioResources.

[4] Wang S., Yu Y., Di M. (2018). Green modification of corn stalk lignin and preparation of environmentally friendly lignin-based wood adhesive. Polymers.

[5] Zhao G., Ni H., Ren S., Fang G. (2018). Correlation between solubility parameters and properties of alkali lignin/pva composites. Polymers.

[6] Xiong F., Han Y., Wang S., Li G., Qin T., Chen Y., Chu F. (2017). Preparation and formation mechanism of size-controlled lignin nanospheres by self-assembly. Ind. Crop. Prod..

[7] Wang Y.-Y., Li M., Wyman C.E., Cai C.M., Ragauskas A.J. (2018). Fast fractionation of technical lignins by organic cosolvents. ACS Sustain. Chem. Eng..

[8] Jiang X., Savithri D., Du X., Pawar S., Jameel H., Chang H.-M., Zhou X. (2017). Fractionation and characterization of kraft lignin by sequential precipitation with various organic solvents. ACS Sustain. Chem. Eng..

[9] Stücker A., Schütt F., Saake B., Lehnen R. (2016). Lignins from enzymatic hydrolysis and alkaline extraction of steam refined poplar wood: Utilization in lignin-phenol-formaldehyde resins. Ind. Crop. Prod..

[10] Prado R., Erdocia X., Labidi J. (2016). Study of the influence of reutilization ionic liquid on lignin extraction. J. Clean. Prod..

[11] Lauberts M., Sevastyanova O., Ponomarenko J., Dizhbite T., Dobele G., Volperts A., Lauberte L., Telysheva G. (2017). Fractionation of technical lignin with ionic liquids as a method for improving purity and antioxidant activity. Ind. Crop. Prod..

[12] Liu E., Li M., Das L., Pu Y., Frazier T., Zhao B., Crocker M., Ragauskas A.J., Shi J. (2018). Understanding lignin fractionation and characterization from engineered switchgrass treated by an aqueous ionic liquid. ACS Sustain. Chem. Eng..

[13] Cheng C., Wang J., Shen D., Xue J., Guan S., Gu S., Luo K. (2017). Catalytic oxidation of lignin in solvent systems for production of renewable chemicals: A review. Polymers.

[14] Abbott A.P., Capper G., Davies D.L., Rasheed R.K., Tambyrajah V. (2003). Novel solvent properties of choline chloride/urea mixtureselectronic supplementary information (ESI) available: Spectroscopic data. Chem. Commun..

[15] Zhang Q., De Oliveira Vigier K., Royer S., Jerome F. (2012). Deep eutectic solvents: Syntheses, properties and applications. Chem. Soc. Rev..

[16] Paiva A., Craveiro R., Aroso I., Martins M., Reis R.L., Duarte A.R.C. (2014). Natural deep eutectic solvents-solvents for the 21st century. ACS Sustain. Chem. Eng..

[17] Ninomiya K., Omote S., Ogino C., Kuroda K., Noguchi M., Endo T., Kakuchi R., Shimizu N., Takahashi K. (2015). Saccharification and ethanol fermentation from cholinium ionic liquid-pretreated bagasse with a different number of post-pretreatment washings. Bioresour. Technol..

[18] Procentese A., Johnson E., Orr V., Garruto Campanile A., Wood J.A., Marzocchella A., Rehmann L. (2015). Deep eutectic solvent pretreatment and subsequent saccharification of corncob. Bioresour. Technol..

[19] Xu G., Ding J., Han R., Dong J., Ni Y. (2016). Enhancing cellulose accessibility of corn stover by deep eutectic solvent pretreatment for butanol fermentation. Bioresour. Technol..

[20] Chen Z., Wan C. (2018). Ultrafast fractionation of lignocellulosic biomass by microwave-assisted deep eutectic solvent pretreatment. Bioresour. Technol..

[21] Lynam J.G., Kumar N., Wong M.J. (2017). Deep eutectic solvents ability to solubilize lignin, cellulose, and hemicellulose; thermal stability; and density. Bioresour. Technol..

[22] Pang K., Hou Y., Wu W., Guo W., Peng W., Marsh K.N. (2012). Efficient separation of phenols from oils via forming deep eutectic solvents. Green Chem..

[23] Alvarez-Vasco C., Ma R., Quintero M., Guo M., Geleynse S., Ramasamy K.K., Wolcott M., Zhang X. (2016). Unique low-molecular-weight lignin with high purity extracted from wood by deep eutectic solvents (DES): A source of lignin for valorization. Green Chem..

[24] Di Marino D., Stöckmann D., Kriescher S., Stiefel S., Wessling M. (2016). Electrochemical depolymerisation of lignin in a deep eutectic solvent. Green Chem..

[25] Li T., Lyu G., Liu Y., Lou R., Lucia L.A., Yang G., Chen J., Saeed H.A.M. (2017). Deep Eutectic Solvents (DESs) for the isolation of willow lignin (*Salix matsudana cv. Zhuliu*). Int. J. Mol. Sci..

[26] Xia Q., Liu Y., Meng J., Cheng W., Chen W., Liu S., Liu Y., Li J., Yu H. (2018). Multiple hydrogen bond coordination in three constituent deep eutectic solvents enhances lignin fractionation from biomass. Green Chem..

[27] Sluiter A., Hames B., Ruiz R., Scarlata C., Sluiter J., Templeton D., Crocker D. (2010). Determination of Structural Carbohydrates and Lignin in Biomass.

[28] Deshpande R., Giummarella N., Henriksson G., Germgård U., Sundvall L., Grundberg H., Lawoko M. (2018). The reactivity of lignin carbohydrate complex (LCC) during manufacture of dissolving sulfite pulp from softwood. Ind. Crop. Prod..

[29] You T., Zhang L., Zhou S., Xu F. (2015). Structural elucidation of lignin-carbohydrate complex (LCC) preparations and lignin from *Arundo donax* Linn. Ind. Crop. Prod..

[30] Min D., Yang C., Chiang V., Jameel H., Chang H. (2014). The influence of lignin-carbohydrate complexes on the cellulase-mediated saccharification II: Transgenic hybrid poplars (*Populus nigra* L. and *Populus maximowiczii* A.). Fuel.

[31] Hong S., Lian H., Sun X., Pan D., Carranza A., Pojman J.A., Mota-Morales J.D. (2016). Zinc-based deep eutectic solvent-mediated hydroxylation and demethoxylation of lignin for the production of wood adhesive. RSC Adv..

[32] Chen L., Dou J., Ma Q., Li N., Wu R., Bian H., Yelle D.J., Vuorinen T., Fu S., Pan X. (2017). Rapid and near-complete dissolution of wood lignin at ≤80 °C by a recyclable acid hydrotrope. Sci. Adv..

[33] Jääskeläinen A., Liitiä T., Mikkelson A., Tamminen T. (2017). Aqueous organic solvent fractionation as means to improve lignin homogeneity and purity. Ind. Crop. Prod..

[34] Zhang S., Liu L., Fang G., Yan N., Ren S., Ma Y. (2017). Hydrogenolysis and activation of soda lignin using [BMIM]Cl as a catalyst and solvent. Polymers.

[35] Kai D., Tan M., Chee P.L., Chua Y.K., Yap Y.L., Loh X.J. (2016). Towards lignin-based functional materials in a sustainable world. Green Chem..

[36] Liu L., Huang G., Song P., Yu Y., Fu S. (2016). Converting industrial alkali lignin to biobased functional additives for improving fire behavior and smoke suppression of polybutylene succinate. ACS Sustain. Chem. Eng..

[37] Wen J., Sun S., Yuan T., Sun R. (2015). Structural elucidation of whole lignin from eucalyptus based on preswelling and enzymatic hydrolysis. Green Chem..

[38] Constant S., Wienk H.L.J., Frissen A.E., Peinder P.D., Boelens R., van Es D.S., Grisel R.J.H., Weckhuysen B.M., Huijgen W.J.J., Gosselink R.J.A. (2016). New insights into the structure and composition of technical lignins: A comparative characterisation study. Green Chem..

[39] Zhao J., Xiu W., Hu J., Liu Q., Shen D., Xiao R. (2014). Thermal degradation of softwood lignin and hardwood lignin by TG-FTIR and Py-GC/MS. Polym. Degrad. Stabil..

[40] Rajić N., Logar N.Z., Rečnik A., El-Roz M., Thibault-Starzyk F., Sprenger P., Hannevold L., Andersen A., Stöcker M. (2013). Hardwood lignin pyrolysis in the presence of nano-oxide particles embedded onto natural clinoptilolite. Microporous Mesoporous Mater..

[41] Malaeke H., Housaindokht M.R., Monhemi H., Izadyar M. (2018). Deep eutectic solvent as an efficient molecular liquid for lignin solubilization and wood delignification. J. Mol. Liq..

[42] Soares B., Tavares D.J.P., Amaral J.L., Silvestre A.J.D., Freire C.S.R., Coutinho J.A.P. (2017). Enhanced solubility of lignin monomeric model compounds and technical lignins in aqueous solutions of deep eutectic solvents. ACS Sustain. Chem. Eng..

[43] Lourenço A., Rencoret J., Chemetova C., Gominho J., Gutiérrez A., Pereira H., del Río J.C. (2015). Isolation and structural characterization of lignin from cardoon (*Cynara cardunculus* L.) stalks. Bioenergy Res..

[44] Del Rio J.C., Prinsen P., Rencoret J., Nieto L., Jimenez-Barbero J., Ralph J., Martinez A.T., Gutierrez A. (2012). Structural characterization of the lignin in the cortex and pith of elephant grass (*Pennisetum purpureum*) stems. J. Agric. Food. Chem..

[45] Azarpira A., Ralph J., Lu F. (2013). Catalytic alkaline oxidation of lignin and its model compounds: A pathway to aromatic biochemicals. Bioenergy Res..

[46] Kim K.H., Dutta T., Ralph J., Mansfield S.D., Simmons B.A., Singh S. (2017). Impact of lignin polymer backbone esters on ionic liquid pretreatment of poplar. Biotechnol. Biofuels.

